# Effect of Acute Ozone Induced Airway Inflammation on Human Sympathetic Nerve Traffic: A Randomized, Placebo Controlled, Crossover Study

**DOI:** 10.1371/journal.pone.0018737

**Published:** 2011-04-08

**Authors:** Jens Tank, Heike Biller, Karsten Heusser, Olaf Holz, André Diedrich, Theodor Framke, Armin Koch, Anika Grosshennig, Wolfgang Koch, Norbert Krug, Jens Jordan, Jens M. Hohlfeld

**Affiliations:** 1 Institute of Clinical Pharmacology, Hannover Medical School, Hannover, Germany; 2 Fraunhofer Institute for Toxicology and Experimental Medicine (ITEM), Hannover, Germany; 3 Division of Clinical Pharmacology, Department of Medicine, Autonomic Dysfunction Center, Vanderbilt University School of Medicine, Nashville, Tennessee, United States of America; 4 Department of Biomedical Engineering, Vanderbilt University School of Engineering, Nashville, Tennessee, United States of America; 5 Institute for Biometry, Hannover Medical School, Hannover, Germany; University Institute of Social and Preventive Medicine, Switzerland

## Abstract

**Background:**

Ozone concentrations in ambient air are related to cardiopulmonary perturbations in the aging population. Increased central sympathetic nerve activity induced by local airway inflammation may be one possible mechanism.

**Methodology/Principal Findings:**

To elucidate this issue further, we performed a randomized, double-blind, cross-over study, including 14 healthy subjects (3 females, age 22–47 years), who underwent a 3 h exposure with intermittent exercise to either ozone (250 ppb) or clean air. Induced sputum was collected 3 h after exposure. Nineteen to 22 hours after exposure, we recorded ECG, finger blood pressure, brachial blood pressure, respiration, cardiac output, and muscle sympathetic nerve activity (MSNA) at rest, during deep breathing, maximum-inspiratory breath hold, and a Valsalva maneuver. While the ozone exposure induced the expected airway inflammation, as indicated by a significant increase in sputum neutrophils, we did not detect a significant estimated treatment effect adjusted for period on cardiovascular measurements. Resting heart rate (clean air: 59±2, ozone 60±2 bpm), blood pressure (clean air: 121±3/71±2 mmHg; ozone: 121±2/71±2 mmHg), cardiac output (clean air: 7.42±0.29 mmHg; ozone: 7.98±0.60 l/min), and plasma norepinephrine levels (clean air: 213±21 pg/ml; ozone: 202±16 pg/ml), were similar on both study days. No difference of resting MSNA was observed between ozone and air exposure (air: 23±2, ozone: 23±2 bursts/min). Maximum MSNA obtained at the end of apnea (air: 44±4, ozone: 48±4 bursts/min) and during the phase II of the Valsalva maneuver (air: 64±5, ozone: 57±6 bursts/min) was similar.

**Conclusions/Significance:**

Our study suggests that acute ozone-induced airway inflammation does not increase resting sympathetic nerve traffic in healthy subjects, an observation that is relevant for environmental health. However, we can not exclude that chronic airway inflammation may contribute to sympathetic activation.

## Introduction

Based on large epidemiological studies, the World Health Organization estimated that air pollution is the 13^th^ leading cause of mortality worldwide.[1] A large proportion of the excess mortality can be attributed to cardiovascular deaths.[2] Long term exposure to fine particles and to ozone were associated with an increased cardiopulmonary mortality.[3] Short term exposure to air pollutants is sufficient to elicit changes in cardiovascular and pulmonary function in healthy subjects[4] and in patients[5]. The rapid onset implicates a neural mechanism. Indeed, autonomic nervous system imbalance with raised sympathetic and attenuated parasympathetic activity may contribute to cardiovascular morbidity and mortality in this setting.[6] Increased ozone and fine particle exposure were associated with attenuated heart rate variability (HRV) and excessive oxidative stress and inflammatory biomarkers in venous blood samples.[7], [8], [9] Ozone exposure reduced HRV in asthma patients [10] but not in otherwise healthy older subjects.[11] Field studies cannot dissect the individual contribution of pollutants on cardiovascular control. The mechanisms through which ozone and fine particle influence autonomic function may differ.[12], [10], [9] Moreover, all previous studies relied on indirect methodologies, such as HRV analysis, to assess autonomic cardiovascular regulation. None employed direct measurements of muscle sympathetic nerve activity (MSNA) or detailed plasma catecholamine analysis. In animals, experimental inflammation in the kidney[13] or in gastrointestinal organs[14] increases central sympathetic activity through afferent neural pathways. Ozone induced airway inflammation [15] could elicit a similar response. A similar mechanism could also contribute to sympathetic activation in patients with chronic obstructive pulmonary disease[16] or asthma.[17] We challenged healthy volunteers with ozone in a double blind, randomized, and cross-over fashion to test the hypothesis that neutrophilic airway inflammation induced by acute ozone exposure is accompanied by increased muscle sympathetic nerve activity.

## Methods

### Participants

Healthy ozone responsive women and men aged 22 to 47 years with forced expiratory volume in the first second (FEV_1_) >80% were eligible for our study. Subjects had to respond to ozone during a screening challenge with 250 ppb ozone over three hours. We defined a response as ≥10% increase in sputum neutrophils 6 hours after the start of ozone exposure. Subjects with respiratory tract infection in four weeks before screening or a positive skin prick test to common aeroallergens were excluded. Subjects regularly taking prescription or over the counter medication except acetaminophen for pain relief, oral contraceptives, hormonal replacement therapy, or vitamin supplements were also excluded.

### Ethics statement

The ethics committee of the Hannover Medical School, Carl Neuberg Str. 1, 30625 Hannover, Germany, approved the study and all patients gave written informed consent.

### Protocol

We exposed healthy ozone responsive subjects in a randomized, double-blinded, cross-over fashion for 3 hours to ozone (250 ppb) or clean air. In the Fraunhofer ozone exposure chamber (2.7×2.3×2.5 m^3^), air temperature and relative humidity were kept in a range of 20–25°C and 40–60%, respectively. A high purity ozone mass flux was generated using commercial generator (COM_ADM, ANSEROS GmbH, Tübingen, Germany). Ozone concentration in the chamber was continuously monitored by two independent analyzers (Ozomat MP, ANSEROS, Tübingen, and 400A, MLU-Messtechnik für Luft und Umwelt GmbH, Essen). During exposure, subjects conducted intermittent bicycle ergometer training for 15 minutes at intensities increasing ventilation to 20 l/min/m^2^ alternating with 15 minutes rest. Sessions were conducted at least 2 weeks apart.

We obtained induced sputum 6 hours after the start of challenge. Subjects inhaled ultrasonically nebulized pyrogen-free hypertonic saline through a mouthpiece while wearing a nose clip. After 5 minutes inhalation, we asked subjects to rinse the mouth and blow the nose to minimize sputum contamination. Then, subjects expectorated into a sterile container. Saline concentration was increased stepwise from 3% to 4% and 5% with expectoration after each step. Sputum was immediately processed. In short, sputum plugs were selected from the whole expectorate (including saliva), and transferred to a pre-weighted cup to determine its weight. To homogenize the sputum, 4 volumes of freshly prepared 0.1% sputolysin (dithiothrietol) was added and incubated for 15 min on a bench rocker. The dispersed sputum was filtered through a 70 µm cell strainer and the cell number was counted by hemacytometer. The differential cell count was assessed on cytospin slides by counting at least 400 non-squamous cells.[18], [19]

Blood samples were obtained from a cubital vein before as well as 5, 7, and 24 hours after start of exposure challenge. Spirometry was performed according to ATS standards before as well as 3, 6, and 24 hours after start of challenge. Cardiovascular and microneurographic measurements were obtained approximately 19 to 22 hours after end of exposure.

### Cardiovascular and sympathetic measurements

We conducted our measurements after an overnight fast in the morning hours. During testing, subjects remained in the supine position. Electrocardiogram, beat-by-beat blood pressure (Finapres, Ohmeda, Englewood, CA, U.S.A.), and brachial blood pressure (Dinamap, Critikon, Tampa, FL, U.S.A.) were determined. We inserted one antecubital venous catheter for blood sampling. Muscle sympathetic nerve activity was recorded from the right peroneal nerve (Nerve Traffic Analyzer 662C-3, Biomedical Engineering Department, University of Iowa, Iowa City, IA, USA) as described previously.[20] Briefly, a tungsten electrode with uninsulated tip (diameter 1–5 µm, shaft diameter 200 µm, 2 Mega Ohm, Frederick Haer and Co, Bowdoinham, MA) was inserted into the muscle nerve fascicles of the peroneal nerve at the fibular head. The raw nerve signal was band-pass filtered (700–2000 Hz), amplified (100×999.9), rectified and integrated (time constant of 0⋅1 s) to obtain mean voltage neurograms (IMSNA) using the nerve traffic analysis system (662C-3, University of Iowa). Satisfactory recordings of MSNA were defined by (1) heart pulse synchronicity; (2) facilitation during Valsalva straining and suppression during the hypertensive overshoot phase after release; (3) increases in response to breath-holding; and (4) no change during tactile or auditory stimulation.

MSNA bursts were identified by an automated detection algorithm with artifact elimination, dynamic noise level detection, and signal-to-noise estimation in the integrated signal. Bursts were accepted if the signal-to-noise ratio was greater than 2:1 and synchronization to a previous cardiac event was found in an interval between 1.2–1.6 seconds. All detections were visually verified.

After a resting period of at least 30 minutes, we obtained baseline recordings over 15 minutes. Then, we obtained blood samples for plasma catecholamine determination with high pressure liquid chromatography. Cardiac output measurements were obtained using an inert gas rebreathing method (Innocor, Innovision A/S, Odense, Denmark).

After baseline measurements had been obtained, we determined cardiovascular responses to fixed breathing at a rate of six breaths per minute in order to reach the highest heart rate variability values in individual subjects.[21] Then, subjects were asked to perform a Valsalva maneuver, by exhaling into a mouthpiece fixed to a mercury manometer with a pressure of 40 mmHg column for 15 sec. Finally, subjects conducted a maximum voluntary inspiratory breath hold maneuver.

Signals were digitized at a sampling rate of 500 Hz and 16 bit resolution using hardware based intelligent oversampling method (DI720USB, DATAQ Instruments, Akron, OH), and then processed with user software written in PV-Wave (Visual Numerics Inc., Houston, TX). We determined the following MSNA parameters from the integrated nerve signal: the burst frequency, i.e. the number of MSNA bursts per minute (bursts/min), the burst incidence, i.e. the number of bursts per 100 heart beats (bursts/100 heart beats), as well as the total activity, i.e. the area under the bursts per minute as arbitrary units per minute (au/min). [20]

Beat-to-beat values of detected R–R intervals and BP values were interpolated, low-pass filtered (cutoff 0.5 Hz) and re-sampled at 4 Hz. Data segments of 300 s were used for spectral analysis. Linear trends were removed and power spectral density was estimated with the FFT-based Welch algorithm using segments of 256 data points with 50% overlapping and Hanning window. The power in the frequency range of low frequencies (LF: 0.04 to 0.15 Hz) and high frequencies (HF: 0.15 to 0.40 Hz) was calculated following Task Force recommendations.[22]

Baroreflex gain was defined as the mean magnitude value of the transfer function between systolic blood pressure and R-R intervals in the low-frequency (BRSLF) and high-frequency (BRSHF) band with negative phase and squared coherence value greater than 0.5. We calculated for our settings (300 sec at 4 Hz, 256 data points segment length, Hanning window) coherence values of 0.33 for p<0.01 and 0.229 for p<0.05. Thus, the coherence limit of 0.5 applied by us and others is suitable for the calculation of baroreflex sensitivity.[23] Spontaneous baroreflex slope was calculated as the slope of the linear regression line between the systolic BP and the subsequent R-R intervals using sequences defined as an episode of at least three heart beats with more than 0.5 mm Hg systolic BP changes and 5 msec R-R interval changes. For sequences with rising (BRSup) and falling BP (BRSdown) the averaged value of slopes with a correlation coefficient greater than 0.85 was calculated.

### Statistical analysis

All data are expressed as mean ± SEM. All parameters were evaluated by a standard cross over analysis with a two-sided t-test adjusting for a potential period effect. Continuous variables were checked for the normal distribution assumption using the D'Agostino and Pearson omnibus normality test. In case of a non Gaussian distribution of the variable the results were confirmed with a non parametric test (Wilcoxon signed rank test) using GraphPad-Prism 5.

For description of the adjusted period effect, means, standard deviations, minimum, maximum and medians for each period and sequence were analyzed. 95% confidence intervals and p-values were calculated. R 2.11.0 has been used for calculations (including packages lattice 0.18–8 and reshape 2.11.0). For the power/sample size calculation, R 2.11.0 and nQuery 7 have been used. A value for p<0.05 was considered significant. Based on the assumption of a relevant treatment difference of 5 bursts/min a sample size of 11 subjects was calculated for the parameter burst frequency at baseline to achieve a power of 80% to detect a difference in measurements after ozone or clean air (two sided t-test, type I error of 5%).

## Results

### Cohort characteristics and disposition

We included fifteen healthy, ozone-responsive subjects in our study (12 men, 3 women, age 22 to 47 years (34 +/− 10 years), body weight 59 to 104 kg (80 +/− 11 kg). One subjects had to be withdrawn due to an upper respiratory tract infection. Two successful nerve recordings of comparable quality could be obtained in 11 subjects. Characteristics of the subjects are shown in table 1.

**Table 1:** Characteristics of the study population.

	Sequence 1 (air -> ozone)	Sequence 2 (ozone -> air)	Total
Number	6	8	14
Male (%)	4 (66.7%)	7 (87.5%)	11 (78.6%)
Age [years]	33.2 (±10.34)	34.1 (±9.5)	33.7 (±9.5)
Weight [kg]	76.7 (±10.7)	83.12 (±11.9)	80.4 (±11.4)
Height [cm]	179.8 (±8.4)	185.6 (±5.7)	183.1 (±7.3)
BMI [kg/m^2^]	23.6 (±1.8)	24.0 (±2.3)	23.9 (±2.0)

Shown are either absolute frequencies and percentages or means and standard deviations.

### Ozone-induced airway inflammation

The percentage of neutrophils in induced sputum obtained 6 hours after the start of challenge (n = 14) was increased after ozone compared to clean air (estimated treatment effect adjusted for period: 16% (difference of percentages), 95% CI: 4.06% to 28.22%, p<0.05), indicating the presence of neutrophilic airway inflammation (table 2). FEV_1_ at baseline was 4.4 +/− 0.6 L before the clean air exposure and 4.4 +/− 0.7 L before the ozone exposure. Forced vital capacity (FVC) at baseline was 5.5 +/− 0.8 L before the clean air exposure and 5.5 +/− 0.7 L before the ozone exposure. Immediately after leaving the challenge chamber, FEV_1_ and FVC were significantly decreased (FEV_1_: estimated treatment effect adjusted for period: −0.37 L, 95% CI: −0.58 L to −0.15 L, p<0.005; FVC: estimated treatment effect: −0.32 L, 95% CI: −0.48 L to −0.16 L, p<0.005). However, 24 hours after the start of challenge, the effect of ozone exposure on lung function parameters was no longer observed (FEV_1_: estimated treatment effect adjusted for period: 0.007 L, 95% CI: −0.07 L to −0.085 L, p = 0.844; FVC: estimated treatment effect: 0.042 L, 95% CI: −0.047 L to −0.131 L, p = 0.321). Airway inflammation was paralleled by signs of systemic inflammation. The percentage of neutrophils in peripheral blood was increased 5 hours after start of ozone challenge compared with clean air (estimated treatment effect adjusted for period: +10.24% (difference of percentages), 95% CI: 5.95% to 14.52%, p<0.0005). At 24 hours after challenge, all changes in the peripheral white blood cell count had recovered to baseline (estimated treatment effect adjusted for period difference of percentages of neutrophils in peripheral blood: −0.964%, 95% CI: −7.993% to 6.064%, p<0.768).

**Table 2:** Differential cell count in induced sputum at baseline and the exposure effect adjusted for period 6 hours after the start of exposure to ozone compared to clean air.

Parameter	Baseline	Treatment effect	95%CI		p-value
Neutros/g	42.2±10.3	91.82±39.7	5,23	178,4	0,0394
Neutros perc	42.4±5.4	16.14±5.54	4,06	28,22	0,0131
Total cells	171.3±46.3	98.38±82.5	−81,4	278,2	0,2563
Total cells/g	84.6±11.4	92.41±47.7	−11,4	196,3	0,0764
Macros perc	25.0±4.0	−11±4.61	−21,1	−0,95	0,0345
Eos perc	0.02±0.02	−0.12±0.06	−0,25	0,01	0,0693
Lymphos perc	1.04±0.19	−0.48±0.44	−0,47	1,43	0,2935
Monos perc	0.79±0.18	−0±0.51	−1,11	1,12	0,9946
Epis perc	30.8±4.6	−5.5±3.12	−12,3	1,3	0,1034
Squamous cells	26.7±4.0	−5.52±4.46	−15,2	4,19	0,2392
Cell viability	58.1±5.5	−5.44±3.73	−13,6	2,7	0,1711

Mean ± SEM, n = 14, CI confidence limits, g = gram, perc  =  per cent, cells/g  =  cells/gram, neutros =  neutrophil granulocytes, macros =  macrophages, eos  = eosinophil granulocytes, lymphos  = lymphocytes, epis  =  epithelial cells.

### Hemodynamics and sympathetic regulation

Resting HR was 59±2 bpm after exposure to clean air and 60±2 after ozone exposure. Resting systolic blood pressure (clean air: 121±3 mmHg; ozone: 121±2 mmHg), diastolic blood pressure (clean air: 71±2 mmHg; ozone: 71±2 mmHg), cardiac output (clean air: 7.42±0.29 mmHg; ozone: 7.98±0.60 l/min), plasma norepinephrine levels (clean air: 213±21 pg/ml; ozone: 202±16 pg/ml), and plasma epinephrine levels (clean air: 23±3 pg/ml; ozone: 23±3 pg/ml) were similar on both study days. No significant estimated treatment effect adjusted for period was detected for HR, BP, CO and plasma catecholamines.

**Figure 1:**
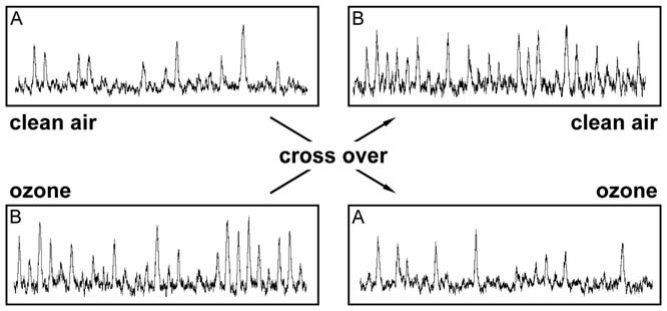
Examples of integrated muscle sympathetic nerve activity recorded at about 19–22 hours after exposure to clean air or to ozone in two subjects.

**Figure 2:**
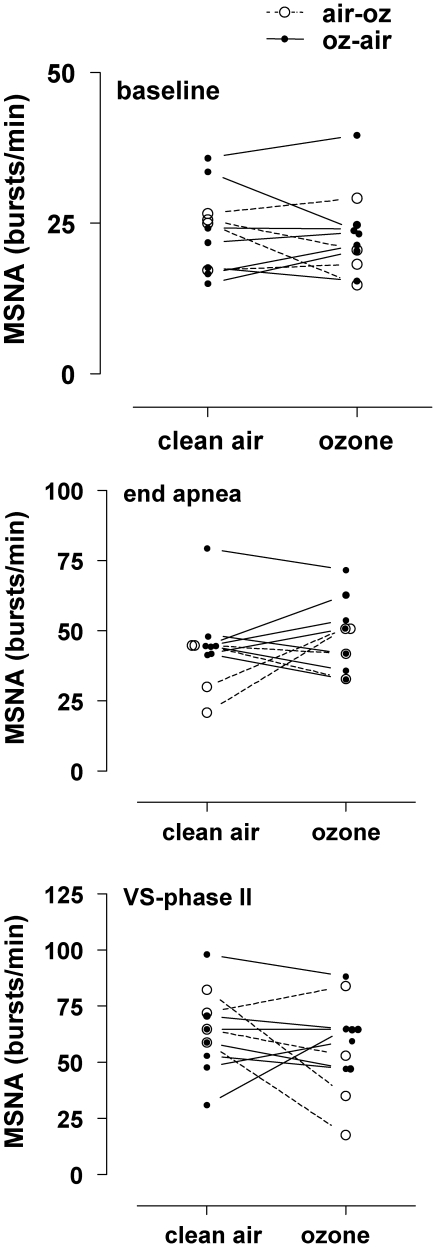
Individual values of muscle sympathetic nerve activity (burst frequency in bursts/min) measured after clean air and ozone exposure at rest (top), at the end of maximum inspiratory apnea (middle) and during late phase II of the Valsalva maneuver (bottom) for subjects exposed first to clean air (empty circles, n = 4) and for subjects exposed first to ozone (filled circles, n = 7).

Figure 1 illustrates sympathetic nerve recordings on both study days for two subjects with different sequence. Muscle sympathetic nerve activity was similar after exposure to ozone and to clean air. Figure 2 shows individual values of muscle sympathetic nerve activity measured after clean air and ozone exposure. Table 3 presents the mean values of sympathetic nerve activity as burst frequency, burst incidence and area under the burst measured on both study days at rest, during deep breathing, at the end of maximum inspiratory apnea and during late phase II of the Valsalva maneuver. No significant estimated treatment effect adjusted for period was detected. MSNA at the end of apnea quantified as burst area tended to be increased with ozone.

**Table 3:** Muscle sympathetic nerve activity (burst frequency, burst incidence and area under the burst) measured on both study days at rest, during deep breathing (6 breaths per minute), at the end of the maximum voluntary inspiratory breath hold maneuver (end apnea) and during late phase II of the Valsalva maneuver.

parameter	clean air	ozone	difference	p-value
***baseline***				
MSNA (bursts/min)	23±2	23±2	−0.68	0.7161
MSNA (bursts/100 beats)	42±5	39±5	−2.69	0.4886
MSNA (area/min)	1.23±0.30	1.04±0.15	−0.14	0.5861
***deep breathing***				
MSNA (bursts/min)	20±2	22±2	1.45	0.4119
MSNA (bursts/100 beats)	33±4	35±4	1.84	0.5322
MSNA (area/min)	1.20±0.28	1.16±0.19	−0.04	0.8802
***end apnea***				
MSNA (bursts/min)	44±4	48±4	5.94	0.1614
MSNA (bursts/100 beats)	68±5	71±4	6.16	0.3127
MSNA (area/min)	3.79±0.45	5.05±0.75	1.5	0.0862
***Valsalva late phase II***				
MSNA (bursts/min)	64±5	57±6	−10.19	0.1496
MSNA (bursts/100 beats)	75±4	68±6	−10.28	0.1293
MSNA (area/min)	3.94±0.36	4.83±0.96	0.74	0.4950

mean values ± SEM (n = 11), MSNA =  muscle sympathetic nerve activity.

### Heart rate variability and baroreflex sensitivity

Table 4 presents the mean values of heart rate variability, blood pressure variability, and baroreflex sensitivity measured on both study days at rest and during deep breathing. All calculated parameters were similar on both study days. No significant estimated treatment effect adjusted for period was detected.

**Table 4:** Heart rate variability (HRV), blood pressure variability (BPV) and baroreflex sensitivity (BRS) data measured on both study days at rest and during deep breathing.

parameter	clean air	ozone	difference	p-value
***baseline***				
***HRV and BPV***				
TP (ms^2^)	2722±424	3433±688	874	0.2212
LF (ms^2^)	1099±241	1363±346	333	0.3132
HF (ms^2^)	540±80	906±287	398	0.0925
LF/HF	2.15±0.34	2.05±0.43	−0.03	0.9343
LF-SBP (mmHg^2^)	8±1	9±2	1.24	0.4831
***BRS (ms/mmHg)***				
BRSup	22±2	26±3	3.89	0.1911
BRSdown	21±3	23±3	1.91	0.5779
BRSLF	13±2	13±2	0.17	0.9306
BRSHF	27±3	30±4	3.25	0.4400
***deep breathing***				
***HRV and BPV***				
TP (ms^2^)	11254±2101	12692±2541	1658	0.2840
LF (ms^2^)	8815±1653	10652±2188	2035	0.1720
LF-SBP (mmHg^2^)	21±3	25±5	4.51	0.2051
***BRS (ms/mmHg)***				
BRSup	35±3	39±4	3.88	0.3087
BRSdown	21±2	19±2	−3.19	0.1657
BRSLF	16±1	16±2	0.20	0.8649

mean values ± SEM, TP =  total power, LF =  low frequency, HF  = high frequency, LF/HF  =  LF to HF ratio, LF-SBP  =  Low frequency power of systolic blood pressure variability, BRSup  = baroreflex sensitivity calculated by the sequence technique for increasing blood pressure, BRSdown  = baroreflex sensitivity calculated by the sequence technique for decreasing blood pressure, BRSLF =  baroreflex sensitivity calculated by cross spectral analysis in the low frequency band, BRSHF =  baroreflex sensitivity calculated by cross spectral analysis in the high frequency band.

## Discussion

The main finding of our study is that neutrophilic airway inflammation induced by acute ozone exposure was not accompanied by changes in muscle sympathetic nerve activity in healthy younger subjects. We combined the microneurography technique with hemodynamic measurements, plasma catecholamine determination, cardiovascular autonomic function testing, and heart rate as well as blood pressure variability analysis, which provides more comprehensive insight in autonomic regulation than either method alone. None of these measurements changed with ozone.

Ozone exposure is an established model to induce neutrophilic airway inflammation. The increase in sputum neutrophil counts observed after ozone challenge in our study is comparable with earlier results.[15], [24] Thus, 250 ppb ozone exposure over three hours in combination with intermittent exercise leads to consistent increases in neutrophilic airway inflammation. We assessed airway inflammation six hours after the start of ozone exposure. Given recent recommendations not to perform more than one sputum induction within 48 hours, we did not obtain additional samples at a later time point.[25] However, other studies applying similar methodology showed that neutrophilic airway inflammation persists for at least 24 hours.[18], [26] In contrast, blood neutrophil counts rapidly normalize after ozone exposure. Thus, our study allowed to study influences of local inflammation in the lung without a major systemic inflammatory response. The issue is important because systemic inflammation could mask sympathetic activation induced by airway inflammation.[27] For ethical reasons we did not obtain airway biopsies for histological analysis. Therefore, we do not have direct evidence that increased neutrophil counts in sputum are associated with inflammation of deeper airway wall portions. However, in previous studies, bronchial biopsies taken 6 hours[28], [29] or 18 hours [30], [31] after an acute ozone exposure in healthy subjects showed increased percentage of neutrophils and total protein concentration in the bronchial fraction. The observation is consistent with acute inflammatory cell influx across airway walls. In one study, inflammatory markers in biopsies did not change with ozone exposure.[29] However, epithelial shedding and substance P release from subepithelial sensory nerves was demonstrated suggesting that mucosal nerve endings are affected by ozone exposure.[32] Furthermore, experiments in anesthetized and artificially ventilated dogs showed ozone responsive bronchial C fibers and rapidly adapting receptors [33]. Chest discomfort, which commonly occurs with deep ozone inhalation, suggest a similar mechanism in human beings. Therefore, we suggest that the design of our study is suitable to assess acute influences of superficial airway inflammation on cardiovascular autonomic control.

In contrast to many other cardiovascular autonomic measurements, resting MSNA exhibits surprisingly little intraindividual variability in healthy subjects.[34] The technique has been proven useful studying interactions between respiratory tract and the cardiovascular system. For example, sympathetic vasomotor tone has been shown to be tightly regulated by chemoreflex mechanisms.[35] Furthermore, MSNA is substantially increased in patients with obstructive sleep apnea [36], [37] and in patients with chronic obstructive pulmonary disease[38]. Sympathetic activity increases profoundly during prolonged breath-holding in some cases about tenfold above baseline.[39] Finally, using the microneurography technique, active smoking and passive smoking were shown to raise sympathetic activity.[40], [41]

Given the excellent reproducibility of the microneurography technique, we are confident that our study excludes a major change in muscle sympathetic nerve activity elicited by ozone-induced neutrophilic airway inflammation in young healthy subjects. In an earlier study, sympathetic vasomotor tone was 34 bursts/min in control subjects and 61 bursts/min in patients with severe chronic obstructive pulmonary disease.[38] Our study was powered to detect 5 bursts/min differences in sympathetic vasomotor tone. Sympathetic reflexes regulating the cardiovascular system often interact with each other as exemplified by baroreflex/chemoreflex interactions.[42] Possibly, sympathetic activation through another reflex pathway could be modulated by neutrophilic airway inflammation. In the event, ozone exposure did not change the sympathetic response to respiratory stimuli like deep breathing, maximum endinspiratory apnea, or the Valsalva-Maneuver. Thus, our study challenges the idea that neutrophilic airway inflammation is sufficient to drive an increase in muscle sympathetic nerve activity in the absence of abnormalities in blood gases or pulmonary hemodynamics. Provided that the acute stimulus was sufficiently strong and properly targeted, our study suggests that airway inflammation does not elicit a major change in muscle sympathetic nerve activity. However, we cannot exclude that more chronic changes in airway inflammation contribute to sympathetic activation in chronic obstructive lung disease or in asthma. As our subjects were healthy, we cannot exclude the possibility that ozone exposure produces or exacerbates sympathetic activation in COPD or asthmatic patients.

Sympathetic nerve activity during apnea quantified as area under the burst tended to be increased with ozone exposure. The observation might suggest that airway inflammation exacerbates sympathetic activation when an additional stimulus is present.

Our observations on experimental ozone exposure are relevant for environmental health, which largely relies on epidemiological observations. Autonomic nervous system imbalance with increased sympathetic and decreased parasympathetic cardiovascular control may be mechanistic link between air pollution and excess cardiovascular mortality.[1] The rapid onset of cardiovascular events after exposure supports this idea.[43] Indeed, in an epidemiological study in 2681 men and women, resting heart rate increased slightly during an air pollution episode.[44] Fine particle exposure during the previous day reduced heart rate variability in another study.[11] Reduced heart rate variability was also observed with increased exposure to ozone.[45] However, the relationship may be attenuated after statistical adjustment for fine particle exposure.[46] Finally, experimental exposure to an ozone/fine particle mixture raised diastolic blood pressure.[47] Changes in autonomic regulation with exposure to air pollution may result from direct effects on rapidly adapting receptors or C-fibers in the lung or oxidative stress and release of inflammatory cytokines in the lung or elsewhere in the body.[43] Our study suggests that persistent airway inflammation induced by acute exposure to ozone without additional pollutants is unlikely to induce an acute change in cardiovascular autonomic regulation. The idea is supported by the observation that environmental ozone exposure is associated with increased risk for death from respiratory causes whereas cardiovascular risk is related to fine particle exposure.[3]

### Conclusion

We applied ozone exposure as a model for neutrophilic airway inflammation, which is a common feature observed in chronic obstructive lung disease. Given the design of our study, we were only able to test the effect of an acute airway inflammation, therefore we cannot exclude that chronic airway inflammation has the potential to affect autonomic cardiovascular regulation. Nevertheless, our study suggests that acute airway inflammation is not sufficient to drive sympathetic activation, an observation that is also relevant for environmental health. Over the last decades, pulmonary medicine and cardiovascular medicine developed exciting new methodologies. Our study underscores the need to join forces for patient oriented research projects on mechanisms mediating cardiovascular disease in pulmonary patients.
